# Factors associated with latrine utilization among model and non-model families in Laelai Maichew Woreda, Aksum, Tigray, Ethiopia: comparative community based study

**DOI:** 10.1186/s13104-018-3683-0

**Published:** 2018-08-13

**Authors:** Gidey Gebremedhin, Desalegn Tetemke, Meresa Gebremedhin, Gizienesh Kahsay, Hiwot Zelalem, Hailay Syum, Hadgu Gerensea

**Affiliations:** 1 School of Public Health, College of Health Science, Aksum University, Aksum, Ethiopia; 2 Public Health Department, College of Health Science, Arsi University, Asela, Ethiopia; 3 School of Nursing, College of Health Science, Aksum University, Aksum, Ethiopia

**Keywords:** Latrine, Model household, Utilization

## Abstract

**Objective:**

The study was conducted on 313 model and 313 non model households to assess latrine utilization and factors affecting among model and non-model families.

**Result:**

About 225 (71.9%) model and 144 (46%) non-model participants declared that they utilize their latrine which gave the overall utilization rate of 369 (58.9%). Households with primary and above education were two times (AOR = 2.03, 95% CI 1.427, 4.638) more likely to utilize latrine as compared with illiterate households. Cleanness of the latrine was also found to be associated with latrine utilization in both model and non-model families. Age, type of latrine, latrine supper structure, cleanness and observable soap near the latrine in model families and age, educational status, occupation, latrine privacy and cleanness in non-model families were identified as a statistical significant factor for latrine utilization.

## Introduction

Lack of sanitation and inadequate hygiene are crucial issues that is associated with disease like diarrhea, cholera, typhoid and parasitic infection [1]. Globally the estimated disease burden associated with poor water, sanitation, and hygiene accounts for 4.0% of all deaths and 5.7% of the total disease burden [2].

About 39% of the world population does not have access to improved sanitation and open defecation is largely a rural phenomenon, most widely practiced in Southern Asian and Sub-Saharan Africa. Sub-saharan countries, including Ethiopia are considered to be the home 81% of open defecation [3–5]. Furthermore, overall Africa’s low access rates to improved sanitation are partly explained by negligible service coverage in rural areas, where the bulk of the population still resides [5, 6].

Nearly 800 million people still do not have access to improved sources of drinking water protected from outside contamination [7, 8].

Even though Ethiopia has achieved greater progress in reduction of open defecation from 93 to 45% at national level and from 100 to 53% in rural areas, there is poor latrine utilization among individual and families in some communities and 38.1 million people still practice open defecation [9, 10]. To solve this problem, the Ethiopian Ministry of Health began health extension program for rural sanitation as part of its mission to extend health care coverage. As a result latrine coverage has improved significantly to 72% across the country and 87% in Tigray region [10]. But latrine utilization is still very low 31% and 34% in Ethiopia and Tigray, respectively.

The main challenges to utilize latrine consistently was often fill quickly [11–13]. Therefore the aim of this study was to assess latrine utilization and factors affecting among health extension model and non-model families in the Woreda.

## Main text

### Methods and materials

#### Study setting and design

The study was conducted in Woreda Laelai Maichew which is located in central zone of Tigray region, northern part of Ethiopia which is 1024 km far away from Addis Ababa the capital city of Ethiopia and 250 km from Mekelle, the capital city Tigray region. The Woreda has four health centers, thirteen health posts and sixteen rural kebeles with a total population of 89,052 [14–23]. The study uses a comparative cross-sectional study design.

#### Sample size and sampling techniques

The sample size was determined by double population proportion formula with the assumptions of; P1 = Proportion of latrine utilization among non-model households = 37.4% (14), P2 = 50% since there was study among health extension model r = 1:1 (r is the ratio of the size of sample 1 to sample 2) CI = 95%, margin of error = 5% at α = 1.96 *β* = power of detect in the study (80%) = 0.84, design effect of 1.5 and 10% non-response rate) [10]. Both model and non model participant households were selected through systematic sampling technique.

#### Study population

All model and non-model households in Laelai Maichew Woreda, Aksum, Tigray, Ethiopia.

*Exclusion* Households who were not available during data collection and not voluntary to participate were excluded.

#### Statistical analysis

Both bivariate and multivariate binary logistic regression analysis was done to identify predictors of latrine utilization among model and non-model household. All the statistical tests were done at 5% level of significance and AOR along with 95% CI was reported. The model was built by stepwise regression technique. The overall model goodness of fit was checked by Hosmer–Lemeshow and the prediction power was checked by receiver-operating characteristic (ROC).

#### Operational definition

Functional latrine: Latrine that provided services at the time of data collection even if the latrine required maintenance [10].
Model household: Are household head that have graduated and certified by local government organs after they took adequate theoretical and practical training for 4 days (96 h) by health extension workers on the 16 basic health extension packages and acquiring enough information about the packages [10].
None model household: None graduated household by health extension workers and not certified by local governmental body [10].

### Result

#### Socio-demographic characteristics

A total of 626 with 313 models and 313 non-model HHs were participated in the study which gives response rate of 100%. About 158 (50.5%) non-model and 147 (47%) model families were found within age ranges of 35–50 years. Two hundred thirty-one (73.8%) model and 215 (68.7%) non-model families participated in the study were males. About 244 (78.0%) model and 229 (73.2%) non-model participants were married and majority of the household’s head was male with 252 (80.3%) and 225 (71.9%) for model and non-model families, respectively. Majority 302 (96.5%) model and 284 (90.7%) non-model participants were farmers. About 168 (53.7%) of the model and 128 (40.9%) non-model families had an average monthly income of 500–1500 in Ethiopian birr while 108 (34.5%) of the model and 143 (45.7%) non-model families had monthly income ranges of < 500 Ethiopian Birr. Almost all the respondents were 618 (98.7%) were orthodox christians in their religion and Tigrean 625 (99.8%) in their ethnicity.

#### Latrine utilization

About 225 (71.9%) model and 144 (46%) non-model participants declared that they utilize their latrine which gives the overall utilization rate of 369 (58.9%). However, fresh feces were observed in the latrine of 147 (46.9%) model and 123 (39.3%) non-model families. About 137 (72.5%) of children’s in model families and 78 (60.0%) in the non-model families start to use latrine after the age 7 years (Table 1).

**Table 1:** Environmental characteristics of model and non-model families in Woreda Laelai Maichew, Tigray, Ethiopia, 2016

Variables	Categories	Model	Non-model	Total (%)
		Frequency (%)	Frequency (%)	
Maintenance	Required	250 (79.9)	272 (86.9)	522 (83.4)
	Not required	63 (20.1)	41 (13.1)	104 (16.6)
Supper structure	No	180 (57.5)	207 (66.1)	387 (61.8)
	With wood	64 (20.4)	59 (18.8)	123 (19.6)
	Plastered with mud	69 (22.0)	47 (15.0)	116 (18.6)
Material used	Cement	35 (11.1)	12 (3.8)	47 (7.5)
	Earth with sand	152 (48.4)	196 (62.4)	348 (55.6)
	Wood with planks	105 (33.4)	93 (29.6)	198 (31.6)
	Mixed	21 (6.7)	12 (3.8)	33 (5.3)
Latrine location	Inside the compound	304 (96.8)	296 (94.6)	600 (95.8)
	Outside the compound	9 (2.9)	17 (5.4)	26 (4.2)
Distance from house (m)	10–15	244 (77.7)	219 (70.0)	463 (74.0)
	16–20	52 (16.6)	67 (21.3)	119 (19.0)
	21–25	16 (5.1)	25 (8.0)	41 (6.5)
	> 25	1 (0.3)	2 (0.6)	3 (0.5)
Privacy	No privacy	151 (48.2)	211 (67.4)	362 (57.8)
	Poor privacy	74 (23.6)	63 (20.1)	137 (21.9)
	Adequate privacy	88 (28.2)	39 (12.4)	127 (20.3)
Cleanness	No	214 (68.4)	254 (81.2)	468 (74.8)
	Yes	99 (31.6)	59 (18.8)	158 (25.2)
Water availability near latrine	No	242 (77.3)	266 (85.0)	508 (81.2)
	Yes	71 (22.7)	47 (15.0)	118 (18.8)
Motivational factors for latrine utilization	Health extension	130 (41.5)	133 (42.5)	263 (42.0)
	Local leaders	63 (20.1)	66 (21.1)	129 (20.6)
	Disease prevention	43 (13.7)	32 (10.2)	75 (12.0)
	Neighbor	19 (6.1)	6 (1.9)	25 (4.0)
	Volunteer community	16 (5.1)	19 (6.1)	35 (5.6)
	Mixed	42 (13.4)	57 (18.2)	99 (15.8)
Supportive supervisory by Woreda health office	No	9 (2.9)	12 (3.8)	21 (3.5)
	Yes	304 (97.1)	301 (96.2)	605 (96.5)
Number of visit by Woreda health office	No observation	9 (2.9)	12 (3.8)	21 (3.5)
	1 times per month	30 (9.6)	34 (10.9)	64 (10.2)
	1–2 times per month	143 (45.5)	129 (41.2)	272 (43.5)
	3–4 times per month	131 (41.7)	138 (44.1)	269 (43.0)
Information on control and prevention of diseases	No	6 (1.9)	31 (9.9)	37 (5.9)
	Yes	307 (98.1)	282 (89.8)	589 (94.1)
Sources of information	Health extension	144 (46.0)	195 (62.3)	339 (57.1)
	From TV	3 (1.0)	8 (2.6)	11 (1.9)
	From members families	10 (3.2)	16 (5.3)	26 (4.3)
	From radio	35 (11.1)	20 (6.4)	55 (9.3)
	Mixed	121 (38.7)	42 (13.4)	163 (27.4)

#### Factors associated with latrine utilization among model and non model households

Households with primary and above education were two times (AOR = 2.03, 95% CI 1.427, 4.638) more likely to utilize latrine as compared with illiterate households. Moreover, farmers were 64.9% (AOR = 0.351 95% CI 0.150, 0.826) less likely to utilize latrine as compared with households who construct pit latrine without slab/earth was four times (AOR = 4.045, 95% CI 1.673, 9.780) more likely to utilize latrine than those who constructed open pit latrine. At the same time households who constructed latrine with slab were 11 times (AOR = 10.769, 95% CI 3.776, 30.708) more likely utilized than those who constructed open pit latrine (Tables 2 and 3).

**Table 2:** Logistic regression analysis of factors associated with Latrine utilization among model families in Woreda Laelai Maichew, Tigray, Ethiopia, 2017

Variable	Category	Latrine utilization		COR (95% CI)	AOR (95% CI)
		Yes (%)	No (%)		
Age	< 35	55 (74.3)	19 (25.7)	2.43 (1.252, 4.721)	2.02 (0.941, 4.329)
	36–50	120 (81.6)	27 (18.4)	3.73 (2.079, 6.703)	4.27 (2.192, 8.318)***
	> 50	50 (54.3)	42 (45.7)	1.00	1.00
Education	Illiterate	107 (64.1)	60 (35.9)	1.00	
	Primary and above	118 (80.8)	28 (19.2)	2.36 (1.406, 3.972)	2.14 (0.538, 4.293)
Type of latrine	Open pit	9 (31.0)	20 (69.0)	1.00	1.00
	Pit without slab	127 (69.4)	56 (30.6)	5.04 (2.160, 11.758)	4.05 (1.673, 9.780)**
	Pit with slab	85 (88.5)	11 (11.5)	17.17 (6.28, 46.98)	10.77 (3.78, 30.71)****
	Vip	4 (80.0)	1 (20.0)	8.89 (0.866, 91.199)	6.04 (0.521, 70.020)
Maintenance requirement	Need maintenance	166 (66.4)	84 (33.6)	1.00	
	Maintained	59 (93.7)	4 (6.3)	7.46 (2.622, 21.246)	
Latrine supper structure	No supper structure	119 (66.1)	61 (33.9)	1.00	1.00
	Only with wood	42 (65.6)	22 (7.0)	0.98 (0.536, 1.785)	0.86 (0.423, 1.738)
	Wood with mud	64 (92.8)	5 (7.2)	6.56 (2.510, 17.154)	4.59 (1.523, 13.846)**
Material used	Mixed	15 (71.4)	6 (28.6)	1.000	
	Earth with sand	99 (65.1)	53 (34.9)	0.75 (0.274, 2.039)	
	Wood with planks	78 (74.3)	27 (25.7)	1.16 (0.407, 3.279)	
	Cement	33 (94.3)	2 (5.7)	6.60 (1.190,36.591)	
Privacy	No privacy	92 (60.9)	59 (39.1)	1.00	
	Poor privacy	55 (74.3)	19 (25.7)	1.86 (1.003, 3.436)	
	Adequate privacy	78 (88.6)	10 (11.4)	5.00 (2.399, 10.432)	
Clean latrine	No	131 (61.2)	83 (38.8)	1.00	1.00
	Yes	94 (94.9)	5 (5.1)	11.91 (4.65, 30.512)	11.91 (4.65, 30.512)****
Observable soap	No	196 (69.5)	86 (30.5)	1.00	1.00
	Yes	29 (93.5)	2 (6.5)	6.36 (1.485, 27.263)	5.58 (1.195, 26.013)*

*p* value * = 0.05–0.01, ** = 0.01–0.001, *** =  < 0.001

**Table 3:** Logistic regression analysis of factors associated with latrine utilization among non-model families in Woreda Laelai Maichew, Tigray, Ethiopia, 2017

variables	Category	Latrine utilization		COR (95% CI)	AOR (95% CI)
		Yes (%)	No (%)		
Age	< 35	45 (51.7)	41 (48.3)	2.820 (1.435, 5.539)	3.112 (1.539, 6.294)**
	36–50	80 (50.6)	78 (49.4)	2.699 (1.462, 4.984)	2.776 (1.464, 5.266)**
	> 50	19 (27.5)	50 (72.5)	1.00	1.00
Education	Illiterate	77 (41.0)	110 (59.0)	1.00	*1.00*
	Primary and above	67 (46.8)	59 (53.2)	1.637 (1.039, 2.58)	2.03 (1.427, 4.638)
Occupation	Other	20 (69.0)	8 (31.0)	1.00	1.00
	Farmer	124 (43.5)	161 (56.5)	0.347 (0.153, 0.788)	0.351 (0.150, 0.826)*
Type of latrine	Open pit	8 (23.5)	25 (76.5)	1.00	
	Pit without slab	99 (45.2)	120 (54.8)	2.681 (1.162, 6.185)	
	Pit with slab	35 (60.3)	23 (39.7)	4.946 (1.910, 12.803)	
	Ventilated latrine	2 (66.7)	1 (33.3)	6.500 (0.519, 81.424)	
Maintenance requirement	Need maintenance	118 (43.2)	154 (56.8)	1.00	
	Maintained	26 (63.4)	15 (36.6)	2.277 (1.155, 4.490)	
Privacy	No privacy	89 (42.0)	122 (58.0)	1.00	*1.00*
	Poor privacy	25 (39.7)	38 (60.3)	0.909 (0.512, 1.614)	0.822 (0.448, 1.508)
	Adequate privacy	30 (76.9)	9 (23.1)	4.607 (2.084, 10.184)	2.942 (1.251, 6.919)*
Latrine cleanness	No	101 (39.6)	153 (60.4)	1.00	*1.00*
	Yes	43 (72.9)	16 (27.1)	4.098 (2.190, 7.667)	4.098 (2.190, 7.667)***

*p* value * = 0.05–0.01, ** = 0.01–0.001, *** =  < 0.001

### Discussion

The overall latrine utilization in this study was 71.9% in model and 46% non model families. The finding is higher as compared to the regional and national reports [10–12] and consistent with [13] compared to non-model families. But lower as compared to the Woreda Laelai Maichew health office report, 80%. This highlighted difference across the region is due to difference in socio economic factors, availability of information, place of residence, reporting system [24, 25].

Latrine utilization among model families was higher than non-model families. Similar study conducted in Souther Nation and Nationality People Republic among model and non-model families also supported our finding [26]. Feces were observed in 147 (46.8%) of the model and 123 (39.3%) of non-model families. Similarly, a study conducted in Shebedino district also revealed that, 99.4% of the households utilize latrine, though; feces were observed by the interviewer around the pit hole in 17.3% of the households [11].

This difference across the region and between model and non-model families were due to the strong supportive supervision of health extension workers, Woreda health office, volunteer community and knowledge of the community related with communicable disease.

Our study also relatively lower as compared to 92% from Hullet Ejjue [27], 92% from Amhara region [11], Denbia 86% [28], as compared to the model families but higher than the study conducted in Awabel 52% [29], Gulomekeda 57.3% [30], Hawzien 37.4% [24], and smaller than non-model families [24]. This difference among model and non-model families are due to the training effect, awareness about latrine utilization and its impact towards prevention of communicable diseases in model families.

The present finding indicated that, even though in every sampled household had their own latrine, they were not utilize the latrine consistently. This situation also supported by findings from a global review demonstrated that owning a latrine does not insure that it is used consistently by household members. 82% in east java, 89% in Kenya [11].

The independent effect of age categories ranges from 36 to 50 years on latrine utilization in model families were four times more likely (AOR = 4.27, 95% CI 2.192, 8.318) compared to the age ranges > 50 years. At the same time age category of 36–50 years were about three times (AOR = 2.736, 95% CI 1.441, 5.193) more likely to utilize the latrine as compared to the age ranges > 50 years as well as age categories < 35 years were three times (AOR = 3.116, 95% CI 1.540, 6.303) more likely utilize the latrine as compared to the age category > 50 years in non-model families. This study is in line with the study conducted in Thailand and Aneded [31–33]. This is due to the fact that, those age categories are active adopter from others, acceptance from role model and also they are active in reading different books as well as they are the middle class to analyze advantages of new services.

As indicated by findings from the presented analysis, education was one of the factors statistically associated with latrine utilization in non-model families. Households with primary and above were two times more likely to utilize latrine as compared to the households with illiterate households (AOR = 2.316, 95% CI 1.083, 4.954). This finding is similar with the study conducted in Hulet Ejju Enessie Woreda, East Gojjam Zone, Amhara Region [27, 33, 34]. This is due to the fact that, this group have the ability to adopt from other role models.

Occupation was also related with latrine utilization in non-model families. Farmers were 65.2% times (AOR = 0.348, 95% CI 0.148, 0.817) less likely to utilize latrine as compared to other occupants. These findings were also consistent with the study conducted in rural Tanzania and Kenya [35]. This is due to the fact that, farmers were passed most of their time far away from their resident house.

Concerning environmental factors, households with pit latrine without slab/earth were four times more likely utilize latrine as compared to those households with open pit latrine in model families (AOR = 4.045, 95% CI 1.673, 9.780). Similarly households with pit latrine and with slab in model families was about 11 times more likely to utilize the latrine than those households with open pit latrine [AOR = 10.769, 95% CI 3.776, 30.708]. This is due to the fact that, open pit latrine creates conducive environment for flies and insects. The present study also consistent with the study conducted in Gulomekeda [AOR = 7.6, 95% CI 3.61–17.10] [30]. This is due to the fact that these two areas are similar in geographical setting, characteristics of the community and method of delivering health information.

Super structure of the latrine was also associated with latrine utilization in the community of the study area. Accordingly model households who constructed latrine using wood plastered with mud were about five times (AOR = 4.592, 95% CI 1.523, 13.846) more likely to utilize latrine as compared to those households who constructed latrine without supper structure. Our results also supported by the study conducted in Awabel district [AOR = 3.008, 95% CI 1.364, 6.631] [29]. This is due to the fact that, super structure play great role on privacy during utilization, protection from rainy during summer season, protection from sun during winter.

Relatively little is known about local perception and cultural barriers for using latrines. Experiences showed that crowding, age, gender, privacy, maintenance of standards, cleanliness, cost, distance and a range of socio cultural economic factors can all affect the acceptability and utilization of latrines either positively or negatively. Our results also show that latrine with adequate privacy were three times (AOR = 2.970, 95% CI 1.262, 6.986) more likely to be utilized compared to the latrine with no privacy in non-model families [32–39].

### Conclusion

Age, type of latrine, latrine supper structure, cleanness and observable soap near the latrine in model families and age, educational status occupation, latrine privacy and cleanness in non-model families were identified as a statistical significant factor for latrine utilization. So that, MOH, regional health bureau and Woreda health office should focus on latrine utilization.

## Limitation of the study

This study was conducted through cross-sectional study and may not show the cause and effect relationship. Moreover, there may be over and under report by the interviewee and have observational bias.

## Abbreviations

CI: confidence interval
AOR: adjusted odd ratio
